# Heparanase and Coagulation–New Insights

**DOI:** 10.5041/RMMJ.10165

**Published:** 2014-10-29

**Authors:** Yona Nadir

**Affiliations:** Thrombosis and Hemostasis Unit, Department of Hematology, Rambam Health Care Campus, Haifa, Israel

**Keywords:** Heparanase, procoagulant, tissue factor, tissue factor pathway inhibitor

## Abstract

Heparanase, a β-D-endoglucuronidase abundant in platelets that was discovered 30 years ago, is an enzyme that cleaves heparan sulfate side chains on the cell surface and in the extracellular matrix. It was later recognized as being a pro-inflammatory and pro-metastatic protein. We had earlier demonstrated that heparanase may also affect the hemostatic system in a non-enzymatic manner. We had shown that heparanase up-regulated the expression of the blood coagulation initiator tissue factor (TF) and interacted with the tissue factor pathway inhibitor (TFPI) on the cell surface membrane of endothelial and tumor cells, leading to dissociation of TFPI and resulting in increased cell surface coagulation activity. Moreover, we have demonstrated that heparanase directly enhanced TF activity which led to increased factor Xa production and subsequent activation of the coagulation system. Recently, heparanase inhibitory peptides derived of TFPI-2 were demonstrated by us to inhibit heparanase procoagulant activity and attenuate sepsis in mouse models.

## INTRODUCTION

Heparanase is an enzyme capable of cleaving heparan sulfate (HS) side chains at a limited number of sites, yielding HS fragments of still appreciable size (∼5–7 kDa).^1^,^2^ Heparanase activity correlated with the metastatic potential of tumor cells, attributed to enhanced cell dissemination as a consequence of HS cleavage and remodeling of the extracellular matrix (ECM) barrier.^3^,^4^ Similarly, heparanase activity was implicated in neovascularization, inflammation, and autoimmunity, involving migration of vascular endothelial cells and activated cells of the immune system.^3^–^5^ Up-regulated expression of heparanase was noted in essentially all human tumors examined, as well as in inflammation, wound healing, and diabetic nephropathy.^3^–^5^ A single human heparanase cDNA sequence was independently reported by several research groups.^6^–^9^ Thus, unlike the large number of proteases that can degrade polypeptides in the ECM, one major heparanase appears to be used by cells to degrade the HS side chains of HS proteoglycans. Expression of heparanase is restricted primarily to the placenta, platelets, and activated blood cells of the immune system, with little or no expression in connective tissue cells and most normal epithelia.^3^,^4^ During embryogenesis, the enzyme is preferentially expressed in cells of the developing vascular and nervous systems.^10^

## HEPARANASE AS A COFACTOR TO TISSUE FACTOR ACTIVITY

Tissue factor (TF) is constitutively expressed in various cell types, including pericytes adjacent to the vessel wall, but absent from the blood cells and endothelial cells surface. This localization is crucial for hemostasis since it prevents a direct contact between TF and the circulating blood. Immunohistochemical studies revealed that many tumors express high levels of TF, including leukemia cells,^11^ raising the possibility of a TF role in the pathogenesis of cancer.^12^ We have demonstrated that heparanase over-expression in human leukemia, glioma, and breast carcinoma cells results in a marked increase in TF levels verified by immunoblot and real-time PCR analyses.^13^ Likewise, TF was induced by exogenous addition of recombinant heparanase to tumor cells and primary endothelial cells, induction that was mediated by p38 phosphorylation and correlated with enhanced procoagulant activity. Induction of TF was further confirmed in heparanase-over-expressing transgenic mice and, moreover, correlated with heparanase expression levels in leukemia patients.^13^ Lately, heparanase was found to exert also non-enzymatic activities, independent of its involvement in ECM degradation and alterations in the extracellular microenvironment.^14^ For example, inactive heparanase enhances Akt signaling and stimulates PI3K- and p38-dependent endothelial cell migration and invasion.^15^ It also promotes vascular endothelial growth factor (VEGF) expression via the Src pathway.^16^ Up-regulation of TF adds another example of the multiple non-enzymatic functions of heparanase. Recently, we have demonstrated that heparanase may serve as a cofactor of TF, suggesting that heparanase is directly involved in activation of the coagulation cascade.^17^ The findings were supported by experiments indicating that heparanase increases the level of factor Xa in the presence of TF/VIIa and the effect is enzymatically independent. The newly generated Xa had the same molecular weight as Xa cleaved by TF/VIIa and was active as depicted by increased conversion of prothrombin to thrombin. Thus heparanase not only up-regulates TF expression in endothelial cells, it also directly interacts with cell surface TF and enhances the generation of factor Xa. Increased Xa generation in the presence of heparanase was shown to be relevant in the clinical setting. Thus, apart from the ability of heparinase to increase Xa levels in normal human plasma, a statistically significant positive correlation was found in patients with acute leukemia and healthy donors between the plasma levels of heparanase and Xa.^17^ Baker et al.^18^ recently reported that heparanase-over-expressing mice generated a larger thrombus within a shorter period of time compared to control mice in arterial injury and stent occlusion models, supporting the procoagulant effect of heparanase.

## HEPARANASE RELEASES CELL SURFACE TISSUE FACTOR PATHWAY INHIBITOR

Tissue factor pathway inhibitor (TFPI) is a plasma Kunitz-type serine protease inhibitor and the only known endogenous modulator of blood coagulation initiated by TF.^19^,^20^ Concentration of TFPI in plasma is increased in patients with acute myocardial infarction.^21^,^22^ There are also reports on increased plasma levels of TFPI in relation to diabetes mellitus,^23^ renal diseases,^24^ and cancer.^25^,^26^ Recently we demonstrated that exogenous addition or over-expression of heparanase by transfected cells resulted in release of TFPI from the cell surface and its accumulation in the cell culture medium.^27^ Importantly, the *in vitro* studies were supported by elevation of TFPI levels in the plasma of transgenic mice over-expressing heparanase. Moreover, increased levels of TFPI have been noted in the plasma of cancer patients,^25^,^26^ reflecting, possibly, induction of heparanase expression and elevation of its plasma levels revealed by ELISA assay.^28^ In human umbilical vein endothelial cell (HUVEC) and tumor-derived cell lines, release of TFPI from the cell surface correlated with enhanced TF-mediated coagulation. This effect was evident already 30 min following heparanase addition, and prior to the induction of TF^13^ or TFPI expression. Thus, heparanase enhances local coagulation activity by two independent mechanisms: induction of TF expression,^13^ and TFPI dissociation from the cell surface. Both functions require secretion of heparanase, but not its enzymatic activity. The underlying mechanism is apparently release of TFPI due to its physical interaction with the secreted heparanase, as clearly evident by co-immunoprecipitation experiments,^27^ reflecting a functional interaction between heparanase and a membrane protein.

Elevated levels of heparanase may be generated locally upon degranulation of neutrophils, mast cells, and platelets,^29^ further facilitating blood coagulation at the site of platelet activation. Hemostatic function of heparanase, executed by inducing TF expression, increasing TF activity, and releasing TFPI from the endothelial cell surface, provides a mechanism by which heparanase contributes to blood coagulation activation.

## NOVEL PEPTIDES DERIVED OF TFPI-2 INHIBITING HEPARANASE PROCOAGULANT ACTIVITY

Tissue factor pathway inhibitor 2 (TFPI-2) and heparanase are two proteins that are present at high levels in the placenta and tumors.^30^–^35^ Recently, we described new peptides derived from the solvent-accessible surface of TFPI-2 that inhibit the heparanase procoagulant activity.^36^ *In vitro*, peptides named 5, 6, 7, 21, and 22, at the length of 11–14 amino acids, inhibited heparanase procoagulant activity but did not affect TF activity. *In vivo*, injection of newly identified peptides 5, 6, and 7 significantly decreased or abolished thrombinantithrombin complex (TAT) plasma levels when heparanase or lipopolysaccharide (LPS) were pre-injected, and inhibited clot formation in an inferior vena cava thrombosis model. Hence, the solvent-accessible surface of TFPI-2 first Kunitz domain was found to be involved in TF/heparanase complex inhibition. The newly identified peptides attenuated activation of the coagulation system induced by heparanase or LPS without predisposing to significant bleeding tendency.^36^

## AN ASSAY TO EVALUATE HEPARANASE PROCOAGULANT ACTIVITY

In order to continue the research of heparanase procoagulant activity we developed a chromogenic assay.^31^ The suggested test is based on the already proven presence of a TF/heparanase complex and the ability of heparins completely to abrogate the complex interaction.^17^ The assay gives information on three parameters: the TF/heparanase complex, TF, and heparanase activities. The assay is easy to perform; results are available in a short time and are reproducible. Apart from heparanase ELISA measuring heparanase antigen,^28^ no other acceptable assays to measure heparanase in plasma exist. The assay was already tested in four hypercoagulable clinical set-ups including women at delivery,^31^ women using oral contraceptives,^37^ patients with lung cancer,^38^ and patients following orthopedic surgery,^39^ showing significant differences among tested groups, as will be described in the following paragraphs. Further research is needed in other setups, enabling generalization of the test’s relevance and widening our understanding of the contribution of heparanase to the hemostatic balance. Use of the assay as part of thrombophilia work-up and correlating results with newly identified heparanase polymorphisms^40^,^41^ are challenging areas requiring further investigation.

## INCREASED HEPARANASE PROCOAGULANT ACTIVITY IN PREGNANT WOMEN AT DELIVERY

As heparanase is abundant in the placenta and estrogen was found to up-regulate heparanase gene expression in human endometrium,^42^ heparanase procoagulant activity and antigen level were studied in pregnant women at delivery.^31^ In 55 plasma samples, heparanase and TFPI levels were evaluated by ELISA and factor Xa, thrombin levels, and antithrombin activity by chromogenic substrates. Thirty-five samples were obtained from third-trimester pregnant women (weeks 36–41) who were in labor or came for appointed elective cesarean section, and 20 control samples were of non-pregnant healthy women. Heparanase procoagulant activity was significantly higher in the plasma of pregnant women compared to non-pregnant ones (*P*<0.005). Relative contribution of heparanase to the TF/heparanase complex activity was significantly higher in the plasma of pregnant compared to non-pregnant women (29% increase, *P*<0.0001). Differences in heparanase procoagulant activity were more prominent than changes in heparanase ELISA levels, TF activity, factor Xa, thrombin, and free TFPI levels. These results suggest a significant contribution of heparanase to the procoagulant state in late third-trimester pregnancy and at delivery.^31^

## HEPARANASE PROCOAGULANT ACTIVITY IS ELEVATED IN WOMEN USING ORAL CONTRACEPTIVES

Estrogen therapy, known to increase the risk of thrombosis, was previously found to up-regulate heparanase expression.^43^ We studied heparanase procoagulant activity *in vitro* and in women using oral contraceptives (OC).^37^ Estrogen receptor-positive (MCF-7) and negative (MDA-231) cell lines were incubated with estrogen, tamoxifen, and ICI—a pure estrogen receptor antagonist. The cells’ medium was evaluated for TF/heparanase complex activity, TF activity, and heparanase procoagulant activity by chromogenic substrate. Plasma samples of 34 healthy women taking OC and 41 control women not on hormonal therapy were investigated. Tissue factor/heparanase activity, TF activity, heparanase procoagulant activity, and factor Xa levels were studied using chromogenic substrate. Heparanase, thrombin-antithrombin (TAT), and D-dimer levels were analyzed by immunoassays. Estrogen and tamoxifen increased heparanase procoagulant activity in the medium of estrogen receptor-positive (MCF-7) cells. Tissue factor/heparanase activity, TF activity, heparanase procoagulant activity, factor Xa level, and D-dimer level were significantly higher in the OC group compared to the control group. The most dramatic difference was observed in heparanase procoagulant activity, reaching a 3.3-fold increase (*P*<0.0001). Levels of heparanase and TAT measured by ELISA did not statistically differ among the study groups. Thus, estrogen increases heparanase procoagulant activity. The findings of the present study suggest a new potential mechanism of hypercoagulability in OC users.^37^

## HEPARANASE PROCOAGULANT ACTIVITY IS ELEVATED AND PREDICTS SURVIVAL IN NON-SMALL CELL LUNG CANCER PATIENTS

Heparanase is implicated in angiogenesis and tumor progression. Heparanase was found to be up-regulated in essentially all human tumors examined. These include carcinomas of the colon,^44^,^45^ thyroid,^46^ liver,^47^ pancreas,^48^,^49^ bladder,^50^,^51^ cervix,^52^ breast,^53^ gastric,^54^,^55^ prostate,^56^ head and neck,^57^,^58^ as well as multiple myeloma,^59^ leukemia, and lymphoma.^60^ Although increased heparanase antigen level in biopsies of cancer patients was previously demonstrated, we evaluated, for the first time, the heparanase procoagulant activity in the plasma of patients with lung cancer.^38^ Sixty-five patients with non-small cell lung cancer at presentation and 20 controls were recruited. Plasma was studied for TF/heparanase procoagulant activity, TF activity, and heparanase procoagulant activity using chromogenic assay and heparanase antigen levels by ELISA. Heparanase antigen levels were higher in the study group compared to control (*P=*0.05). Tissue factor/heparanase activity and, even more apparent, heparanase procoagulant activity were significantly higher in the study group compared to controls (*P=*0.008, *P<*0.0001, respectively). No significant difference was observed in the TF activity between the groups. A heparanase procoagulant activity higher than 31 ng/mL predicted a mean survival of 9±1.3 months, while heparanase procoagulant activity of 31 ng/mL or lower predicted a mean survival of 24±4 months (*P=*0.001). Heparanase procoagulant activity was higher than 31 ng/mL in the four cases of thrombosis detected during the follow-up period. Hence, elevated heparanase procoagulant activity in patients with lung cancer reveals a new mechanism of coagulation system activation in malignancy. Heparanase procoagulant activity can potentially be used as a predictor for survival.

## INCREASED HEPARANASE LEVEL AND PROCOAGULANT ACTIVITY IN ORTHOPEDIC SURGERY PATIENTS RECEIVING PROPHYLACTIC DOSE OF ENOXAPARIN

Patients undergoing hip and knee replacement surgery require effective thromboprophylaxis, and anticoagulants and mechanical methods have become standard therapies. However, despite prophylaxis, subclinical deep-vein thrombosis develops in approximately 15%–20% of patients shortly after surgery, and symptomatic venous thromboembolism develops in 2%–4% of patients during the first 3 months post-surgery.^61^ We have recently reported a significant increase in heparanase procoagulant activity observed following orthopedic surgery.^39^ The study group included 50 orthopedic patients: 31 patients underwent hip surgery, and 19 had knee operation. Fifteen individuals suffered from traumatic hip fractures, and 35 had osteoarthrosis of hip or knee joints. All patients received prophylactic enoxaparin at a dose of 40 mg, starting 6–8 hours post-operation and lasting for 5 weeks. Plasma samples were drawn preoperatively and at 1 hour, 1 week, and 1 month after surgery. Samples were tested for heparanase levels by ELISA and TF/heparanase complex activity, TF activity, heparanase procoagulant activity, factor Xa, and thrombin levels using chromogenic substrates. The results revealed that heparanase levels were significantly higher at 1 hour and 1 week postoperatively compared to preoperative levels (*P*< 0.05, *P*<0.005, respectively). The most dramatic changes were observed in heparanase procoagulant activity reaching a 2-fold increase 1 week postoperatively and a 1.7-fold increase 1 month after surgery (*P*<0.0001, *P*<0.0001, respectively). Levels of factor Xa and thrombin increased at 1 month post-surgery, similar to the pattern of TF activity, although statistical significance was reached only in factor Xa. According to the results, heparanase is involved in coagulation activation of orthopedic surgery patients. Heparanase procoagulant activity is highest at 1 week post-surgery and remains high 1 month after operation.

## CONCLUSION

Heparanase was recently revealed as an important modulator of blood coagulation.^13^,^17^,^18^,^27^ Generating peptides inhibiting heparanase procoagulant activity^36^ strengthens the role of heparanase as a new procoagulant factor in the coagulation cascade. In order to augment understanding of heparanase we lately developed an assay to evaluate heparanase procoagulant activity in the plasma,^31^ enabling further extensive research in the field. Taking into account the pro-metastatic and pro-angiogenic functions of heparanase, over-expression in human malignancies, and abundance in platelets and placenta, its involvement in the coagulation machinery is an intriguing novel arena for further research.

### TAKE-HOME MESSAGE:

- Heparanase enhances TF activity in the coagulation cascade.
- Heparanase up-regulates TF expression.
- Heparanase releases TFPI from the cell surface.
- Peptides derived of TFPI-2 inhibit heparanase procoagulant activity.
- Heparanase procoagulant activity is elevated in clinical set-ups of known hypercoagulability state

## Abbreviations:

ECM: extracellular matrix;
HS: heparan sulfate;
LPS: lipopolysaccharide;
OC: oral contraceptives;
TAT: thrombin-antithrombin;
TF: tissue factor;
TFPI: tissue factor pathway inhibitor;
VEGF: vascular endothelial growth factor.
